# Do gaze behaviours during action observation predict interpersonal motor resonance?

**DOI:** 10.1093/scan/nsaa106

**Published:** 2020-08-12

**Authors:** Soukayna Bekkali, George J Youssef, Peter H Donaldson, Jason He, Michael Do, Christian Hyde, Pamela Barhoun, Peter G Enticott

**Affiliations:** Cognitive Neuroscience Unit, School of Psychology, Deakin University, Burwood 3125, Australia; Cognitive Neuroscience Unit, School of Psychology, Deakin University, Burwood 3125, Australia; Centre for Adolescent Health, Murdoch Children’s Research Institute, Melbourne 3052, Australia; Cognitive Neuroscience Unit, School of Psychology, Deakin University, Burwood 3125, Australia; Cognitive Neuroscience Unit, School of Psychology, Deakin University, Burwood 3125, Australia; Russell H. Morgan Department of Radiology and Radiological Science, The Johns Hopkins University School of Medicine, Baltimore, MD 21205–2196, USA; Department of Forensic and Neurodevelopmental Sciences, Sackler Institute for Translational Neurodevelopment, Institute of Psychiatry, Psychology, and Neuroscience,King s College London, London WC2R 2LS, UK; Cognitive Neuroscience Unit, School of Psychology, Deakin University, Burwood 3125, Australia; Cognitive Neuroscience Unit, School of Psychology, Deakin University, Burwood 3125, Australia; Cognitive Neuroscience Unit, School of Psychology, Deakin University, Burwood 3125, Australia; Cognitive Neuroscience Unit, School of Psychology, Deakin University, Burwood 3125, Australia

**Keywords:** interpersonal motor resonance, mirror neuron system, gaze behaviours, TMS, eye-tracking

## Abstract

Interpersonal motor resonance (IMR) is a common putative index of the mirror neuron system (MNS), a network containing specialised cells that fire during both action execution and observation. Visual content inputs to the MNS, however, it is unclear whether visual behaviours mediate the putative MNS response. We aimed to examine gaze effects on IMR during action observation. Neurotypical adults (*N =* 99; 60 female) underwent transcranial magnetic stimulation, electromyography, and eye-tracking during the observation of videos of actors performing grasping actions. IMR was measured as a percentage change in motor evoked potentials (MEPs) of the first dorsal interosseous muscle during action observation relative to baseline. MEP facilitation was observed during action observation, indicating IMR (65.43%, SE = 11.26%, *P* < 0.001). Fixations occurring in biologically relevant areas (face/hand/arm) yielded significantly stronger IMR (81.03%, SE = 14.15%) than non-biological areas (63.92%, SE = 14.60, *P* = 0.012). This effect, however, was only evident in the first of four experimental blocks. Our results suggest that gaze fixation can modulate IMR, but this may be affected by the salience and novelty of the observed action. These findings have important methodological implications for future studies in both clinical and healthy populations.

## Introduction

The human mirror neuron system (MNS) is a network of brain regions thought to contain specialised cortical cells (i.e. ‘mirror neurons’) that fire during both the execution and observation of an action, and is hypothesised to provide a simulation capability at the neural level (Rizzolatti and Craighero, 2004). First reported in area F5 of the premotor region in the macaque monkey, the homologous human MNS putatively consists of the inferior frontal gyrus (IFG), inferior parietal lobule (IPL; Rizzolatti and Craighero, 2004), as well as the supplementary motor area (Mukamel *et al.,* 2010). Support for these regions is derived from neuroimaging and electrophysiological techniques that indicate these regions show increased activation during both action execution and observation (Rizzolatti and Craighero, 2004). In terms of the cortical hierarchy of the MNS, visual sensory information enters through the visual cortex and is processed by the superior temporal sulcus (STS). This information is then transferred via anatomical connections to the IPL, where kinaesthetic aspects of observed actions are processed and then forwarded to the IFG, where goals of actions are coded for and circulated back to the STS (Carr *et al.,* 2003). The STS is considered an associated region of the MNS that is critical for processing biological motion, but unlike the IFG and IPL, does not actually contain mirror neurons (Carr *et al.,* 2003) (Kilner and Lemon, 2013).

A common putative neurophysiological index of MNS activity is ‘interpersonal motor resonance’ (IMR), which broadly reflects ‘mirrored’ activation of the motor system during the observation of another’s actions (Uithol *et al.,* 2011). IMR can be measured using transcranial magnetic stimulation (TMS), a non-invasive neuromodulation technique whereby electrical current is induced in the brain by passing magnetic pulses through the skull (Pascual-Leone, 1999). Briefly, TMS administered over the primary motor cortex (M1) produces a muscle response (referred to as a motor evoked potential [MEP]), which, when measured via electromyography (EMG), can be used to index corticospinal excitability (CSE; Fadiga *et al.,* 1995; Strafella and Paus, 2000). When this method is implemented during observation of visual stimuli illustrating execution of the same target muscle, there is typically an increase in cortical excitability (i.e. increased MEP). This motor facilitation during action observation relative to a control condition (e.g. during the observation of a fixation cross), putatively reflects MNS activity occurring in the adjacent premotor cortex and SMA (which then projects to M1), and is referred to as ‘IMR’ (Fadiga *et al.,* 1995; Strafella and Paus, 2000; Gangitano *et al.,* 2001).

IMR has additional functional properties, including temporal dynamics and sensitivity to specific visual properties. It has been previously shown that the IMR response follows the temporal dynamics of the observed movement (i.e. the reaching and grasping of an object) and is thus a reflection of the visual processing of action (Gangitano *et al.,* 2004; Lepage *et al*., 2010a,b). Additionally, it has been suggested that IMR does not occur during the observation of non-biological movement, indicating specificity and sensitivity of the putative MNS to biological information (Lepage *et al.,* 2010b).

The MNS is implicated in many social cognitive domains, including empathy and theory of mind (Iacoboni and Dapretto, 2006; Kaplan and Lacoboni, 2006; Oberman *et al.,* 2007; Schulte-Rüther *et al.,* 2007; Enticott *et al.,* 2008; Moore and Franz, 2016; Bekkali *et al.,* in press). However, the extent of its role in social cognition remains unclear and controversial; current understandings are largely theoretical, and empirical outcomes remain mixed (Bekkali *et al.,* in press). An important consideration not extensively examined when investigating the role of the MNS in social cognition is the potential effect of visual and perceptual factors. As the human MNS receives direct inputs from the visual cortex via the STS, IMR may be influenced by visual gaze factors, such as attention and fixation patterns. Visual information provides extensive non-verbal information about one’s social environment, such as others’ ocular movements and direction/s of attention, facial expressions, body language, and social cues (Frischen *et al.,* 2007; Itier and Batty, 2009). Collectively, these inform social interactions and allow inferences to be made about the mental states of others, which can facilitate appropriate socioemotional responses and promote inter-relational functioning. In other words, visual information is imperative for social cognitive processes and this visual content is the initial input to the MNS via the STS, suggesting that lower level perceptual and visual factors (e.g. gaze behaviours) may influence the putative mirror response.

Few studies, however, have directly investigated the effects of gaze behaviours on mirror neuron activity or IMR. Maranesi *et al.* (2013) showed visual gaze behaviours influence MNS activity of the macaque monkey (area F5) during free observation of grasping actions, where fixating directly on the grasping action resulted in stronger neuronal firing rates relative to fixating elsewhere. Similar findings have been reported in human studies and suggests an effect of gaze behaviours on the putative MNS response, where significant positive associations between IMR and fixation count on biomotion areas of interest (AOIs) have been reported (Donaldson *et al*., 2015). Although these studies provide some evidence for an effect of gaze behaviours on IMR, they are limited by small sample sizes, non-human samples (e.g. Maranesi *et al.,* 2013), and in some cases, a lack of concurrent eye-tracking (e.g. Donaldson *et al*., 2015).

In the current study, we aimed to examine the effects of gaze behaviours (such as fixation location) on IMR during the observation of dynamic grasp actions using TMS and concurrent eye-tracking, which provides a real-time index of visual processing in an unobtrusive and sensitive manner (Liversedge and Findlay, 2000; McCormick *et al.,* 2012). We examined various stimulation timepoints in order to obtain a broader representation of IMR across the action sequence and to investigate the specific phase of movement at which the strongest IMR would occur (Gangitano *et al.,* 2004; Lepage *et al*., 2010a,b). It was hypothesised that the observation of action would elicit a larger MEP (reflecting IMR) relative to the observation of no action (i.e. baseline). It was also hypothesised that gaze behaviours would influence IMR, whereby fixations occurring in biological and action-related interest areas (i.e. hand, face, and arm regions) at the time of MEP measurement would elicit larger IMR.

## Methods

### Participants

Data examined in the current study were part of a larger study examining the neurobiological basis of empathy, which consisted of two face-to-face sessions held at Deakin University (Melbourne Burwood campus). This larger project comprised 159 healthy adults. The current study comprised a subsample of 136 participants who agreed to undergo both TMS and eye-tracking assessment. Participants who were not right handed, aged outside of 18–40 years, or had a positive neuropsychiatric history (as measured by the Mini International Neuropsychiatric Interview screening tool; Lecrubier *et al.,* 1997) were excluded from this project. Participants were also screened for TMS contraindicators with standard exclusionary criteria (Rossi *et al*., 2009). Of the 136 eligible participants, 37 participants were excluded from analysis because: (1) TMS was terminated due to unexpected safety concerns arising during the session (e.g. fatigue and/or TMS-related discomfort or anxiety; *n* = 23; 16.91%) or (2) acquisition/equipment failures of TMS (*n* = 9; 6.61%) or eye-tracking (*n* = 5; 3.67%) during data collection.

**Fig. 1:**
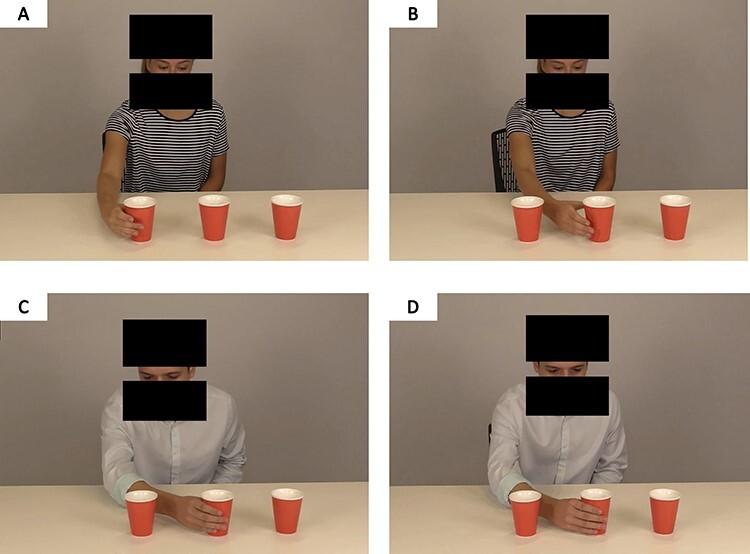
Visual depiction of an action execution sequence presented during the task. (A) Incongruent female; (B) congruent female; (C) incongruent male and (D) congruent male. The blocks in images displayed here have been added to de-identify actors and were not present during the task.

The final sample thus comprised 99 (60 female, 39 male) neurotypical adults aged 18–40 (*M*_age_ = 23.84, SD = 4.81). According to the Edinburgh Handedness Inventory (Oldfield, 1971), all participants were right handed (Oldfield, 1971  *M*_laterality quotient_ = 93.04, SD = 14.51, where a score of 50+ indicates right handedness), and the average number of years of formal education was 16.07 (SD = 2.12, range = 18–40 years). Participants were reimbursed with AUD$40 department store vouchers (AUD$20 per session) for their time.

### Materials

#### Transcranial magnetic stimulation and electromyography

Single pulse TMS was administered using a Magstim 200 stimulator (The Magstim Company Limited, Whitland, UK) and a 70 mm figure-of-eight coil (d70). EMG was recorded using Powerlab 4/35 (AD Instruments, Colorado Springs, CO) with dual BioAmp, via self-adhesive unipolar surface electrodes placed over the first dorsal interosseous (FDI; belly-tendon montage), abductor digiti minimi (ADM; belly-tendon montage) and ulnar styloid process (ground electrode) of the right hand. EMG muscle responses were read on LabChart v8 (AD instruments, Colorado Springs, CO) at a sampling rate of 10 KHz.

#### Biological motion stimulus videos

**Fig. 2:**
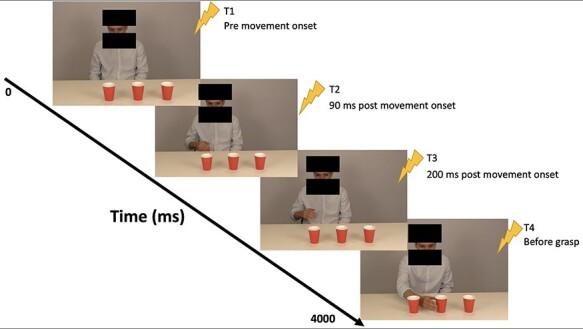
Visual depiction of stimulation (TMS) timepoints delivered during observation of action. The blocks in images displayed here have been added to de-identify actors and were not present during the task.

We developed and recorded videos depicting human actors (one male and one female) executing simple hand grasping actions (Figure 1). Overall, there were nine variations of action sequences in total, each of which were presented by each of the actors. Trials consisted of both ‘congruent’ (*N*_trials_ = 96) and ‘incongruent’ (*N*_trials_ = 36) conditions based on the actor’s eye-gaze and subsequent hand reaching action (see Supplementary Figures SIII and SIV). That is, trials depicted an actor gazing at one of three available cups, who then either grasped a cup that was consistent with the direction of their eye-gaze (congruent) or grasped a cup that was inconsistent with their eye-gaze (incongruent; see Figure 1). For congruent conditions, each trial had four TMS stimulation timepoints (triggered at: 1—prior to movement; 2–90 ms post-movement; 3–200 ms post-movement and 4- at ‘before grasp’, which was kinematically determined), while incongruent trials had three stimulation timepoints (all but ‘prior to movement’; see Figure 2). These timepoint-pulses were automatically triggered using a light-sensor device that detected a white screen embedded in the stimulus videos (not visible to participants), and were selected based on previous findings (Gangitano *et al.,* 2004; Lepage *et al*., 2010a,b).

There were 132 trials in total (see Supplementary Figure SII), presented in four blocks of 33 trials, totalling ~16 min (4 min per block with a brief break in between). Trial order was randomised. Each video displayed for 4000 ms, with an inter-stimulus interval of 3000 ms (black screen). Stimulus videos were programmed using Experiment Builder (SR Research, Ontario, Canada). Participants observed the stimulus videos on a 24″ LED computer screen and were seated ~600 mm away from the screen.

#### Eye-tracking

Right eye-tracking data were acquired using the Eyelink 1000 plus eye-gaze tracking system (with remote mode) at a sampling rate of 500 Hz (SR Research, Ontario, Canada). Eye-tracking was acquired separately for each trial. Target stickers were placed on the centre of the participant’s forehead, and camera set up and calibrations were conducted using a 5-point calibration followed by a prosaccade task where participants were required to visually attend to the moving fixation cross. Gaze error was maintained at <0.5° and drift corrections were performed consistently between each of the four blocks. Participants who typically wore glasses were able to keep them on during the experiment.

### Procedure

TMS was first employed to determine the 1 millivolt (mV) resting motor threshold (RMT). To determine RMT, the coil was held tangentially over the left primary motor cortex (M1) at a 45° angle to the mid-sagittal line. Coil angle and stimulation intensity were adjusted until the lowest intensity evoking MEPs averaging ~1 mV in the contralateral hand over 10 consecutive trials was established; this was the RMT (Hyde *et al.,* 2018). The average RMT of participants was 51.43% of maximum stimulation output (SD = 10.68, range = 28–80%). Using the predetermined individual RMT, participants then received 20 pulses delivered 5000 ms apart while observing a fixation cross, which served as our pre-stimulus baseline index of CSE.

Next, participants passively observed the biological stimulus videos while receiving TMS at the four time-points specified above: (T1) immediately prior to movement onset of the actor; (T2) 90 ms after movement onset; (T3) 200 ms after movement onset and (T4) just prior to hand grasp (where the ‘grasp’ timepoint was approximated manually for each video; see Figure 2; Gangitano *et al.,* 2004; Lepage *et al*., 2010a,b). Each trial consisted of one of the four possible stimulation TPs, and the order of pulse delivery was randomised. The time point of delivery, condition type (congruent *vs* incongruent), and sex of the actor were randomised and eye-tracking was measured concurrently. Following stimulus videos, participants received a second baseline (post-stimulus videos) identical to the pre-baseline condition (i.e. 20 pulses delivered 5000 ms apart while observing a fixation cross). A post-baseline condition, which was administered ~2–3 min following the fourth and final block of the experiment, was included to assess if CSE changed over the course of the experiment and to control for the potential effects of repetitive stimulation across time.

## Data analysis

### Eye-tracking data

**Fig. 3:**
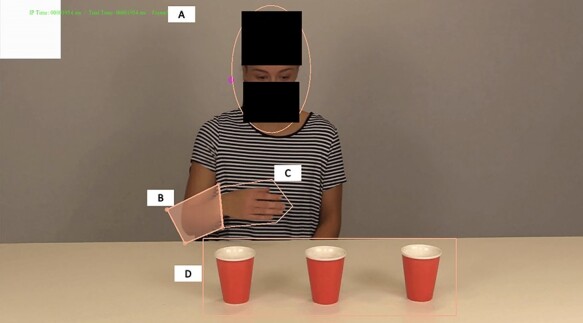
Interest area locations: (A) face, (B) arm, (C) hand and (D) cups. The blocks in images displayed here have been added to de-identify actors and were not present during the task.

All eye-tracking data were pre-processed using Eyelink Data Viewer (SR Research, Ontario, Canada). Four dynamic AOIs (areas of interest) were drawn around the following regions of interest (see Figure 3): face (circular), arm (freehand), hand (freehand) and cups (rectangular; freehand was used when the actor’s hand interacted with the cups). These AOIs were selected as they are important for transitive action-execution and biological motion, which are critical to the generation of IMR (Fadiga *et al.,* 1995; Donaldson *et al*., 2015). Categories of specific AOIs were created in order to examine their effects on IMR (see Table 1). These categories consist of AOIs that reflect similar visual properties, such as biological AOIs (i.e. hand, arm and face), or action–execution AOIs (i.e. hand and arm; see Table 1). Additionally, for fixations occurring outside of these AOIs, we computed a variable termed ‘elsewhere’. These were considered ‘inattentive’ trials.

**Table 1:** Area of Interest (AOI) sub-categories

	Categories					
	Biological		Action execution		Attention	
AOI	Biological	Non-biological	Action	Non-action	Attentive	Not- attentive
Hand	X		X		X	
Arm	X		X		X	
Face	X			X	X	
Cup		X		X	X	
Elsewhere		X		X		X

Only three stimulation timepoints (T2 [90 ms], T3 [200 ms] and T4 [‘grasp’]) were of interest for the current study and therefore included in the final analyses. T1 (stimulation delivered prior to movement onset) was not analysed given the actor was ‘pre-movement’ and had not yet begun the action–execution sequence (i.e. the hand and arm were not in view; see Figure 2). Finally, it should be noted that, due to a technical error in data collection, incongruent trials were not able to be included in the final analysis. As mentioned earlier, the current data were extracted from a larger study, whereby the neurobiological basis of empathy was investigated, and incongruent trials were only included to provide a predictive coding context. Though this condition was not a variable of interest in the current study, we nonetheless conducted sensitivity analyses to statistically examine whether the removal of these incongruent trials would influence our outcomes. We found that there was no significant main effect of congruency on IMR and the effect size suggested a negligible effect (χ^2^ [1, *N* = 99] = 0.05, *P* = 0.817), and thus, we elected to remove these trials.

### Electromyography data

EMG data were pre-processed and extracted in LabChart Reader v8 (AD Instruments, Colorado Springs, CO). As we assessed IMR in the FDI, we thresholded according to the FDI muscle and did not use the ADM data. Furthermore, activation of the ADM data was generally small and not considered reliable. Raw peak-to-peak MEP amplitudes during experimental and baseline conditions were extracted for the FDI muscle. IMR was then calculated by converting these FDI MEP values (during the experiment and baseline) into a percentage change variable using the following formula:

\begin{equation*} \mathrm{IMR}=\frac{\mathrm{MEP}\kern0.5em \mathrm{action}\ \mathrm{observation}\kern0.5em -\kern0.5em \mathrm{MEP}\kern0.5em \mathrm{baseline}}{\mathrm{MEP}\;\mathrm{baseline}}\times 100 \end{equation*}

where ‘IMR’ is the individual MEP percentage change from baseline per trial, ‘MEP action observation’ is the median raw MEP (FDI) value per trial during the observation of the biological motion stimulus videos, and ‘MEP baseline’ is the median of the 20 MEP (FDI) values acquired during the pre-baseline condition. The median values were used in the current study because it has been previously shown that MEP amplitudes are initially large and decline over time (Schmidt *et al.,* 2009). Due to this transient inflation, we used each participants’ median MEP when calculating the IMR as it is a more reflective measure of central tendency given the data. This IMR computed variable was our measure of MNS activity (i.e. MEP change from baseline) and subsequently used to interpret our results. Additionally, we used the pre-stimulus baseline, as opposed to a combination of both pre and post, since the post-stimulus baseline yielded greater MEPs (*M* = 1.47, SE = 0.06) than the pre-baseline (*M* = 1.17, SE = 0.06, *P* < 0.001) condition. Furthermore, MEPs during post-baseline were not different to MEPs observed during the experimental task (*M* = 1.53, SE = 0.06, *p* = 0.080), indicating there was no change in CSE over time (see Supplementary Figure SI). Considering this, we believe the pre-baseline was more reflective of a true and uncontaminated baseline, given it occurred before the observation of the biological motion stimuli, and was therefore used in calculating IMR.

### Statistical methods

Analyses were conducted using Stata 13 (StataCorp, 2013). In order to account for within-subject correlations across repeated measures, we used linear mixed-effects regression models with participant ID as a ‘by-subject’ random effect. In a series of analyses, we systematically examined whether IMR was related to (1) experimental block (4 levels: block one *vs* two *vs* three *vs* four); (2) stimulation timepoint (3 levels: T2 [90 ms] *vs* T3 [200 ms] *vs* T4 [‘grasp’]); (3) overall gaze fixation behaviour (5 levels: hand *vs* arm *vs* face *vs* cups *vs* elsewhere; see Table 1) and (4) fixation AOIs groupings (see Table 1). In a separate series of analyses, the interactions between each of these different factors were also examined. Additionally, given the large number of repetitive trials presented (and the potential reduction in salience across time), we also tested for attenuation in IMR across blocks (1–4; see below).

Given muscle contractions are known to facilitate TMS-evoked MEPs, we extracted root-mean-square (RMS) for activity occurring within 200 ms of the TMS pulse delivery and accounted for this in each analysis. Data screening revealed the IMR data were skewed and non-normal, so we explored whether applying a natural log transformation, which corrected skew, impacted the interpretation of results. Specifically, we ran our analyses using both the raw (i.e. skewed) and log-transformed data for comparison and found the results to be consistent (see Supplementary Table SI). We therefore present the results of the raw data, as it is easier to interpret the MEP percentage change from baseline (i.e. IMR) as a measure of effect size. We also present the results using the log-transformed data in Supplementary (Table SI).

## Results

### Main analyses

There was evidence for increased IMR percentage change from baseline during action observation (IMR = 65.43%, SE = 11.26%, *P* < 0.001). This result was robust to adjustment for RMS activity, stimulation timepoint, and overall gaze fixation behaviour (65.43%, SE = 11.27%, *P* < 0.001).

**Fig. 4:**
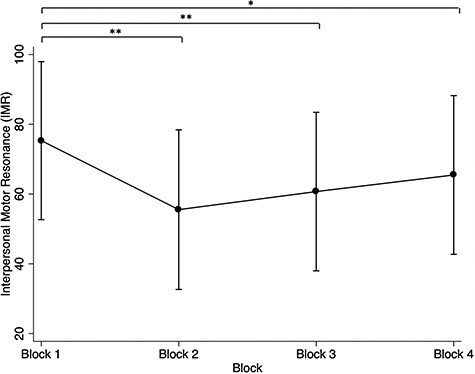
IMR (reflected as the MEP percentage change), with confidence intervals, over blocks. Note: *P < 0.05; **P < 0.001.

Preliminary analyses assessed whether there were differences in IMR across experimental blocks. Overall, there was a main effect of block, χ^2^ (3, *N* = 99) = 18.49, *P* = 0.0003, suggesting that even though the blocks were identical (except for within block trial/condition randomisation), IMR changed over blocks. As shown in Figure 4, pairwise comparisons revealed the IMR in block one (IMR = 75.31%, SE = 11.57) was larger than IMR in block 2 (IMR = 55.51%, SE = 11.69, *P* < 0.001), block 3 (IMR = 62.65%, *P* = 0.005) and block 4 (IMR = 65.48%, *P* = 0.031). No other block differences in IMR were observed. Accordingly, we controlled for ‘block’ in all subsequent analyses. However, given IMR was greatest in block 1, we decided to specifically explore the effects of gaze and stimulation timepoint in this block alone and present the results in a subsequent section. It should be noted that block 1 alone consisted of 18 trials.

Overall, IMR (reflected as the MEP increase during action–observation from baseline) was maintained after accounting for the block effect (IMR = 65.20%, SE = 11.27%, *P* < 0.001).

#### Effect of stimulation timepoint on IMR

**Fig. 5:**
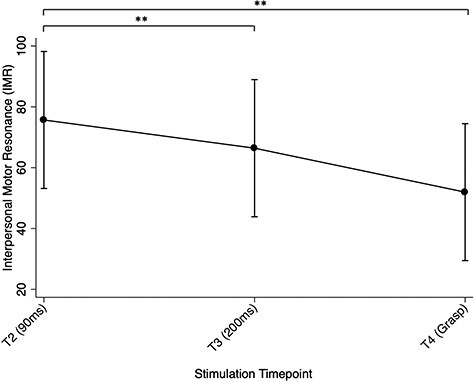
Effect of stimulation timepoints on IMR (reflected as the MEP percentage change), with confidence intervals. Note: *P < 0.05; **P < 0.001.

When assessing the effect of stimulation timepoint (T2 [90 ms], T3 [200 ms] and T4 [‘grasp’]) on IMR, an overall main effect was found, χ^2^(2, *N* = 99) = 37.70, *P* < 0.0001. While all stimulation timepoints resulted in IMR (i.e. increased MEPs during action observation, relative to baseline), results also showed a decrease in IMR across timepoints (T2 [90 ms], T3 [200 ms] and T4 [‘grasp’]; see Figure 5). Pairwise comparisons revealed MEPs at T4 (i.e. ‘grasp’; IMR = 51.95%, SE = 11.48%) were lower than T3 (i.e. 200 ms; IMR = 66.40%, SE = 11.40%, *P* < 0.0001) and T2 (i.e. 90 ms; IMR = 75.68%, SE = 11.48%, *P* < 0.0001; see Figure 5), with T2 yielding the highest IMR relative to baseline. There was no evidence for any interaction effects.

#### Effect of gaze behaviours on IMR

We then assessed the effect of overall gaze fixation behaviours (i.e. hand *vs* arm *vs* face *vs* cups *vs* elsewhere) on IMR and the effects of specific AOI groupings (e.g. AOI: biological *vs* non-biological on *IMR*). Results did not show any main effects for overall gaze behaviours or for any of the relevant AOI groupings (see Table 1), suggesting gaze fixation did not modulate IMR. There were also no significant interactions between overall gaze behaviours, AOIs groupings and stimulation timepoint or block.

### Block one analysis

Given the reduction of IMR over time and the evidence suggesting block 1 elicited the strongest IMR, we repeated the above analysis for block 1 MEPs alone.

#### Effect of stimulation timepoint on IMR

**Fig. 6:**
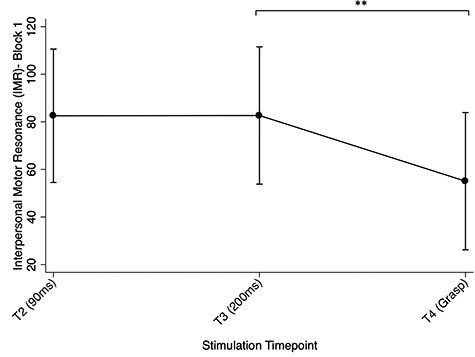
Effect of stimulation timepoints on IMR, with confidence intervals, in block one alone. Note: *P < 0.05; **P < 0.001.

Similar results for stimulation timepoint were found in block 1. There was evidence for an overall main effect (χ^2^ [2, *N* = 99] = 16.99, *P =* 0.002). There was also evidence that T4 (IMR = 55.05%, SE = 14.71%) remained weaker than T2 (IMR = 82.55%, SE = 14.71%, *P* < 0.0001) and T3 (IMR = 82.66%, SE = 14.72%, *P* = 0.001). However, there was no longer evidence for a difference between T2 and T3 (*z* = 0.02, *P* = 0.987; see Figure 6).

#### Effect of gaze on IMR

For overall gaze fixation behaviours, there was some evidence for an effect on IMR, χ^2^ (4, *N* = 99) = 8.38, *P* = 0.078. Pairwise comparisons showed that fixating on the hand region (IMR = 90.20%, SE = 16.23) resulted in higher IMR compared to fixating ‘elsewhere’ (IMR = 67.81%, SE = 15.27) (*P* = 0.048) and fixating on the cups (IMR = 58.56%, SE = 16.16) (*P* = 0.010). Additionally, fixating on the actor’s face (IMR = 78.26%, SE = 14.36) also yielded greater IMR than fixating on the cups (*P* = 0.047). No other differences were revealed.

**Fig. 7:**
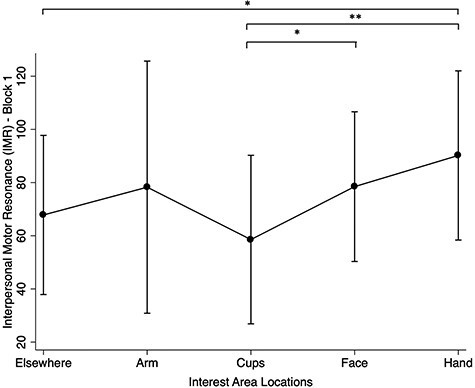
IMR (reflected as the MEP percentage change), with confidence intervals, across fixation locations. Note: *P < 0.05; **P < 0.001.

In block 1, there was also an effect of fixation type, whereby fixation on biological areas (i.e. hand + arm + face) elicited higher IMR (IMR = 81.03%, SE = 14.15%) than non-biological areas (i.e. cups + elsewhere; IMR = 63.92%, SE = 14.60; *P* = 0.012; see Figure 7). For areas relevant to action–execution, there was weak evidence against the null hypothesis, where the observation of action-execution areas (i.e. hand + arm) elicited a larger IMR (IMR = 89.21%, SE = 15.87) relative to non-action execution areas (i.e. face + cups + elsewhere; IMR = 72.27%, SE = 14.00) (*P* = 0.060). There was no significant main effect for attentive *vs* non-attentive AOIs. Further, no interactions were found between stimulation timepoint and gaze behaviours (χ^2^ [8, *N* = 99] = 4.37, *P =* 0.833), fixation type (χ^2^ [2, *N* = 99] = 2.05, *P =* 0.395), fixation movement (χ^2^ [2, *N* = 99] = 2.26, *P =* 0.323) or fixation attention (χ^2^ [2, *N* = 99] = 2.23, *P =* 0.327).

## Discussion

The current study aimed to investigate the relationship between visual gaze behaviours and IMR (reflected in MEP-PC from baseline) during the observation of dynamic grasping actions. Here, we provide some evidence to suggest that IMR, or motor ‘mirroring’, is modulated by gaze behaviours, primarily in earlier trials and during the observation of novel actions, which is consistent with the proposed underlying neural network (i.e. visual cortex projecting to STS, with subsequent projections to sensorimotor regions). In line with recent studies (Maranesi *et al.,* 2013; Donaldson *et al*., 2015; Leonetti *et al.,* 2015), it was hypothesised that stronger MEP-PC from baseline (i.e. IMR) would be observed when subjects’ gaze was fixated on stimulus aspects most relevant to biological motion processing, such as the hand, face and arm, relative to fixations occurring elsewhere. This hypothesis was not supported overall. Across all blocks, fixation locations had no effect on IMR, suggesting specific/central fixation location may not be critical to IMR*.* However, after further investigation, we found gaze behaviours did modulate IMR when salience was highest (i.e. in block one alone where IMR responses were greatest). Below, we discuss the effects of gaze fixation on IMR, considering both the overall and block one findings, followed by the temporal dynamics of these results. We then highlight the limitations of this study and their implications.

### The effect of gaze behaviours on IMR (block one)

Although the effect of gaze behaviours overall (i.e. across all blocks) was not evident, further investigation in block one alone (given the significant attenuation of MEPs across four blocks) revealed fixation location/s did indeed seem to be important for IMR and ‘gaze-dependent’, at least in the initial trials. Specifically, fixations occurring in the hand and face AOIs resulted in significantly higher IMR compared to fixating ‘elsewhere’ and on the ‘cups’. Furthermore, there was weak evidence for an effect of biological (hand + face + arm) *vs* non-biological (elsewhere + cups) fixations, where IMR was stronger during biological fixations. We also observed a trend toward an effect of fixations occurring in regions associated with action execution (hand + arm *vs* other; *p* = 0.060) on IMR. These results, while limited to the first block, are generally consistent with our hypothesis. This suggests IMR was gaze dependent in the first block, where the stimuli were still novel and action prediction was more ambiguous. In other words, when information is new and attention is presumably heightened, visual details are observed in the central foveal field and the features of these specific fixations affect IMR. Direct fixations occurring in biological and ‘action-related’ areas of the stimulus elicit stronger IMR, which is consistent with current methodological understandings of priming IMR and the selective nature of mirror neurons (Uithol *et al.,* 2011).

Although the observed IMR across all blocks seemed to be unaffected by gaze behaviours, it is possible that findings were influenced by repeated exposure to the action observation stimuli. Considering the gaze-dependent fixation processing occurred earlier in the experiment (i.e. in block one), and given the large number of consecutive trials presented in the current study, it is more than possible that the novelty of the stimuli was not maintained over time and resulted in gaze-independent fixation processing occurring in later trials. That is, it is reasonable to assume reduced salience over time leads to reduced attention and more peripheral processing and top–down predictive processing in later blocks (and subsequently, gaze independence), as opposed to central fixations occurring in block one where the novelty of the stimulus was high. It is equally likely that reduced salience resulted in fatigue, boredom and distraction, thus affecting IMR across later blocks. The presentation of numerous identical hand-action sequence videos was considered methodologically necessary in order to collect sufficient and ample data in order to counteract the high variance inherent in TMS data (Magistris *et al.,* 1998; Julkunen *et al.,* 2012). However, this may have resulted in reduced attention and alertness across time, given that no effects of fixation location were found in the main analyses, affecting the IMR across blocks (which was not the case in block one alone when salience was high). It has been shown that, in addition to the known inter-trial variability of TMS data, MEP amplitudes across trials are time variant and can be affected by the number of blocks in an experimental design, resulting in inter-block variability (Julkunen *et al.,* 2012).

The decreased effect of IMR across multiple repetitive trials and blocks has been previously documented in neuroimaging research, where neural activity (reflected in blood oxygen-level depended responses) is diminished after repeated presentations of stimuli (Henson, 2003). Commonly referred to as ‘repetition suppression’ (RS; Mayrhauser *et al.,* 2014), this reduction is thought to occur when the difference in sensorimotor outcomes between what is expected and what is observed (i.e. prediction error) is reduced, which naturally occurs when similar stimuli are consecutively repeated (Friston, 2005; Mayrhauser *et al.,* 2014). That is, when the salience of a stimulus is high (i.e. when it is first presented), there is a peak in prediction error due to high ambiguity and novelty. However, as the stimuli are repeated multiple times, salience is diminished, prediction error is reduced (due to learnt regularities), and neuronal activity is consequently weakened (Friston, 2005; Mayrhauser *et al.,* 2014). RS and the notion of minimising prediction error is consistent with the predictive coding account for mirror neuron processing, where the brain acts as a ‘hypothesis tester’, constantly receiving and updating sensory input and integrating information with stored knowledge gained through prior experience (Friston, 2005; Mayrhauser *et al.,* 2014; Urgen and Miller, 2015). Our block one results are consistent with this perspective, in that the ambiguity of the stimuli would have been high given its novelty, which is reflected in the significantly stronger IMR occurring in the first 33 trials in block one relative to the smaller changes observed in subsequent blocks where the salience of the stimulus would have been diminished. Related to this, it is also likely that learned visuomotor expectations were formed through repetitive observations, which may have been sufficient to engage the motor system via associative learning mechanisms. That is, via the proposed simulation properties of the MNS and Hebbian associative learning resulting from repeated observations, IMR can be generated without the need for constant and direct fixations. This is consistent with current theoretical understandings of associative learning and mirror neuron functioning, where bidirectional associations are forged through learned experiences and drawn upon to predict sensorimotor outcomes (Heyes and Ray, 2000; Heyes, 2012).

### The effect of gaze behaviours on IMR overall (all blocks)

As described earlier, our overall results revealed IMR was not affected by gaze behaviours across all blocks, indicating the effects present in block one were not maintained across the entire experiment. It is possible the observed changes in MEPs from baseline perhaps became ‘gaze-independent’ and no longer contingent upon whether or not participants directly fixated on relevant interest areas of the action sequence. Indeed, there are findings that suggest mirror neuron activation can be elicited in the absence of full visual information during action observation. For instance, Maranesi *et al.* (2013) used single-cell recordings in macaque monkeys during free-gaze observation of grasping actions. The discharge of almost half of the recorded neurons were ‘gaze-independent’ (where neural firing rates did not vary based on the monkey’s direct observation of the specific actions), while the other half were ‘gaze-dependent’. It was suggested that gaze-independent and gaze-dependent neurons form two distinct populations of mirror neurons, where gaze-independent neurons are able to automatically code for actions without directly attending to the stimulus (Maranesi *et al.,* 2013). It is worth noting, however, that gaze-independence does not imply that no relevant visual information enters the visual processing system. In fact, it is suggested that this population of neurons are activated by movement occurring in the peripheral aspects of the visual field (specifically, at ~ >9° from the monkey’s point of central fixation; Maranesi *et al.,* 2013; Leonetti *et al.,* 2015). Although it is understood that the perception of action and biological movement is most kinematically accurate in the central visual field due to high visual acuity, it has been shown that information entering the peripheral visual field is sufficient to elicit motor activation/resonance, and provides general, ‘low-resolution’ information about the observed movement, as opposed to more specific and ‘high-resolution’ visual details that are provided in central foveal vision (Duchowski, 2007; Leonetti *et al.,* 2015).

Previous studies have also shown mirror neuron activation occurs even when crucial parts of the observed action sequence are concealed (e.g. behind a screen). For instance, observing an actor/agent reaching towards a target object is sufficient in eliciting activation, even when the hand–object interaction (which is considered imperative for eliciting a response) is occluded from sight, further suggesting that a population of mirror neurons that are able to code for action in the absence of visible action, which might explain the current findings (Umilta *et al.,* 2001). These results may also be explained by the ‘perception–action’ matching properties and anticipatory simulation capabilities of mirror neurons (Urgen and Miller, 2015), which are thought to underpin IMR. That is, observations of the initial phases of movement may be sufficient to prime prior sensorimotor experiences and pre-existing internal motor models, leading to predictive IMR and action understanding without directly observing the execution of the goal (or, relevant areas of an action sequence) at all times (Maranesi *et al.,* 2013).

Collectively, these studies suggest that visual information detected within the peripheral visual field, coupled with past perceptual history, can be sufficient in eliciting activation without the need for continuous and direct foveal fixation. This is comparable to the conditions used in this study where participants were exposed to multiple trials of almost identical action sequences across four blocks and, given the diminished salience over time, may have drawn upon their own motor repertoire and memory of past trials (as well as inner motor representations of previous motor experiences of action execution), resulting in peripheral visual processing, or, ‘gaze-independence’ (Keysers *et al.,* 2003; Maranesi *et al.,* 2013).

Overall, our gaze-behaviour on IMR results indicate that when information is novel and ambiguity is high, there is a peak in the magnitude of IMR (i.e. MEP-PC) that is gaze-dependent; that is, gaze that is fixated on aspects most relevant to biological motion appears to enhance IMR. Conversely, when the ambiguity and salience of stimulus decrease due to repeated presentations, so too does IMR magnitude and dependence on gaze behaviours, as internal models and stored knowledge are increasingly recruited. It is unclear how these findings can translate to practical applications where multiple trials are required for sufficient data collection. It is clear that further research aimed at defining the ‘threshold’ regarding usefulness of additional trials would be beneficial. Our results highlight the importance of ecologically valid approaches to stimulus presentations and encourage the need for more realistic, complex, and interesting stimuli that would not only maintain engagement, but also be more reflective of the complex interactions we observe in real-life settings. That is, in real world interactions and in day-to-day social situations, we do not encounter the exact repetition of action sequences presented in these experimental settings, and the unrealistic nature of these conditions have important implications for our results.

### Fast IMR processing

We also examined the temporal dynamics of IMR, with the aim of investigating where the strongest changes in MEPs from baseline would occur. Our results showed IMR was strongest 90 ms post-movement onset and declined over the last two stimulation timepoints. Our results are suggestive of earlier motor resonance processing, where the immediate appearance of the hand and onset of movement was not only sufficient in eliciting an IMR response, but also resulted in the strongest IMR. Our results conflict with some previous findings that have reported different temporal dynamics. For instance, Nishitani and Hari (2000, 2002) investigated the temporal sequence of activation across the classical MNS sites during the observation of movement using magnetoencephalography (MEG). Results of these studies consistently showed a longer sequence of activation occurring in succession through visual regions, IPL, IFG and ending in M1 at ~335 ms, suggesting peak latency in M1 occurs much later in post-stimulus time (Nishitani and Hari, 2000, 2002). This contrasts with our results, suggesting that perhaps an alternative and more direct route of information transmission may exist, where motor-mapping mechanisms bypass these classic regions of the MNS (Lepage *et al*., 2010a,b). Previous evidence for a fast motor resonance mechanism during action observation has been provided by neurophysiological research. For instance, a MEG study used lateralised readiness fields (LRF) during action observation and found LRF to occur 83 ms after movement onset (van Schie *et al.,* 2008). Similar results were also reported in a previous TMS study, where enhanced corticospinal activity during action observation was observed between 60 and 90 ms post-movement initiation (Lepage *et al*., 2010a,b). These conflicting findings suggest the possibility that there may be different functional processes of the MNS, and has lead authors to postulate IMR may rely on two mechanisms: (1) a low-level (or ‘crude’), but fast form of IMR that codes action/s automatically, and, in contrast, (2) a higher-level and controlled mechanism of motor processing that requires a slower transmission time for more refined and complicated action understanding. Given the unvaried and repetitive nature of the stimulus used in the current study, it is not unlikely that a lower level, automatic, and basic ‘perception-action matching’ process was recruited, as opposed to a more evaluative, higher level cognitive mechanism which may have required complex forms of action understanding and slower computation (van Schie *et al.,* 2008). However, it should be noted that the current study was limited to one muscle (FDI), and so muscle specificity cannot be determined.

Regarding our block one analysis, the stimulation timepoint effects originally observed across all blocks were largely maintained, with the exception of timepoints two (90 ms post-movement onset) and three (200 ms post-movement onset), which were no longer significantly different. Given the novelty of the stimuli in block one, it is possible more time for information processing was required (reflecting bottom-up processing), resulting in similar IMR across the early (90 ms) and mid (200 ms) timepoints after movement initiation. However, these results still suggest a relatively fast motor processing route, considering the last timepoint (grasp) remained as the stimulation timepoint eliciting the weakest IMR overall.

Collectively, our results supplement the findings of previous research and add weight to the notion that IMR may rely on two distinct forms of processing; where simple observations of action–execution sequences only require low-level and automatic rapid processing as opposed to longer and time consuming processing necessary for more complicated forms of action and information, such as intricate social situations and interactions. Nevertheless, much remains to be learned about the temporal sequences of IMR and varying levels of complexities in regard to action understanding.

### Limitations and conclusions

The results of the current study extend understandings of the temporal dynamics of IMR, but also highlight the potential ramifications of repetitive presentations of simple grasping actions on the magnitude of IMR across time, as well as the effects on visual gaze behaviours during action observation. In addition to these aforementioned limitations and the variability associated with TMS data discussed earlier, eye-tracking technology has limitations worthy of consideration. In particular, the qualitative methods used to determine and draw interest areas, where specificities (location, size, and shape) of AOIs are subjectively determined can be considered potentially problematic (Hessels *et al.,* 2016). Additionally, researcher-defined AOIs in many eye-tracking studies, including the current, are constructed manually (i.e. hand drawn), as opposed to machine made using customised software, which contributes to inconsistencies and variations across studies using similar protocols and stimuli (e.g. simple grasping action videos; Hessels *et al.,* 2016). There are currently no general guidelines or universal consensus on how these AOIs can be constructed, making comparisons across studies difficult and perhaps contributing to the somewhat inconclusive findings within the literature. Discussions around evaluation and refinement of AOI construction methods currently used are necessary, not only for between-study comparisons, but also for valid and reliable outcomes and interpretations within studies. An additional limitation for the current study that should be considered pertains to the lack of ADM data. Although not a muscle of interest here, interpretations of our results should be made with some degree of caution, particularly when making comparisons with other studies that indeed included control muscles. The current study also observed increased CSE during the post-baseline condition relative to the pre-baseline and experimental condition. While unexpected, this finding suggests possible neurophysiological carry-over effects from the observation of the biological action–execution stimuli, resulting in a residual effect in our post-baseline, and rendering this condition unusable. Importantly, it is also imperative to acknowledge that, given the current study did not include additional ‘non-human’ control conditions, it is difficult to distinguish IMR (i.e. MEP facilitation) from other aspects of motor processing, such as motor preparation and motor imagery. Though the experimental design adopted here is entirely consistent with previous work and is considered a sound approach, this limitation is nonetheless a drawback of most IMR studies and important to consider when interpreting the current findings. A final limitation worthy of mentioning pertains to the removal of timepoint one (T1; pre-movement onset) from the analysis. It is entirely plausible that T1 may have offered valuable insights into the effects of gaze orientation prior to movement onset and its potential effects on IMR. However, given that the actor in this condition had not yet began the action–execution sequence (that is, the hand and arm regions were not in view, see Figure 2), this condition was not comparable to other timepoints (where all AOIs were in view), and thus was not able to be included in the final analyses.

## Conclusions

In summary, there was no overall association between gaze behaviours (i.e. fixation location) and IMR across the entire experiment. However, the effects of gaze fixation can modulate IMR when the actions observed are salient and novel, as evidenced by the results of block one. Our study has important implications for future research, particularly in terms of methodological considerations regarding the number of repetitive trials used, and also highlights the need to develop understandings around the construction of AOIs in eye-tracking studies using similar stimuli. Further, given the likely effects of salience on gaze behaviours and IMR, the extent of this relationship remains unclear and warrants further investigation. Future studies aiming to develop and implement more ecologically valid, novel and continuously engaging stimuli should consider routinely administering concurrent eye-tracking in order to elucidate the potential effects of eye gaze behaviours on IMR, without the confound of salience or visual attentional factors. Our findings also have practical implications for studies investigating IMR in psychiatric populations. Given that gaze behaviours are known to differ between clinical groups (e.g. autism spectrum disorders; Richer and Coss, 1976; Riby and Hancock, 2008; Nakano *et al.,* 2010) and controls, the use of concurrent eye-tracking would provide valuable insights into how IMR may be affected by these differences in oculomotor behaviours in clinical populations.

## Conflicts of interest

None declared.

## Funding

P.G.E. is supported by a Future Fellowship from the Australian Research Council (FT160100077), and S.B. was funded by the Deakin University Postgraduate Research Scholarship (DUPR).

## Supplementary Material

nsaa106_Supp
