# Supplementary material for: Do gaze behaviours during action observation predict interpersonal motor resonance?
Source: Soc Cogn Affect Neurosci. 2020 Aug 12;17(1):61–71. doi: 10.1093/scan/nsaa106 (PMC8824634; doi:10.1093/scan/nsaa106)
Supplement: nsaa106_Supp [file nsaa106_supp.zip › scan-19-312-File002_nsaa106.docx]

**Appendix A –Supplementary Material**

**Figure I.** Changes in motor evoked potentials (MEPs) across conditions.

**Figure II.** Trial-type information. Gaze direction indicates the placement of the observed cups.

**Figure III.** Visual depiction of condition types: congruent.

**Figure IV.** Visual depiction of condition types: incongruent.

**Table 1.** Comparison of IMR between non-normal data and log transformed data.


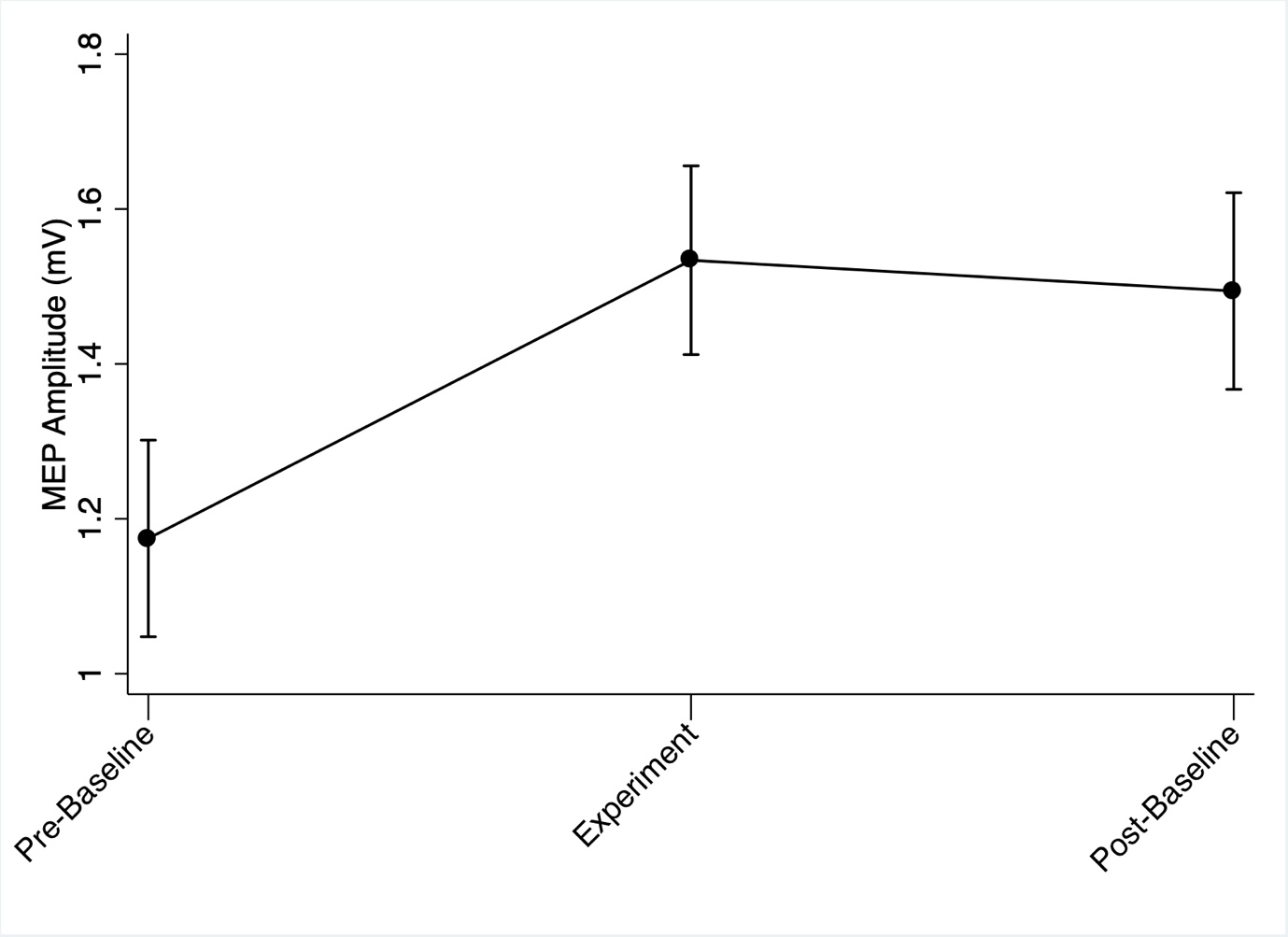


*Figure I.* Changes in motor evoked potentials (MEPs [Mv]) across conditions.

*
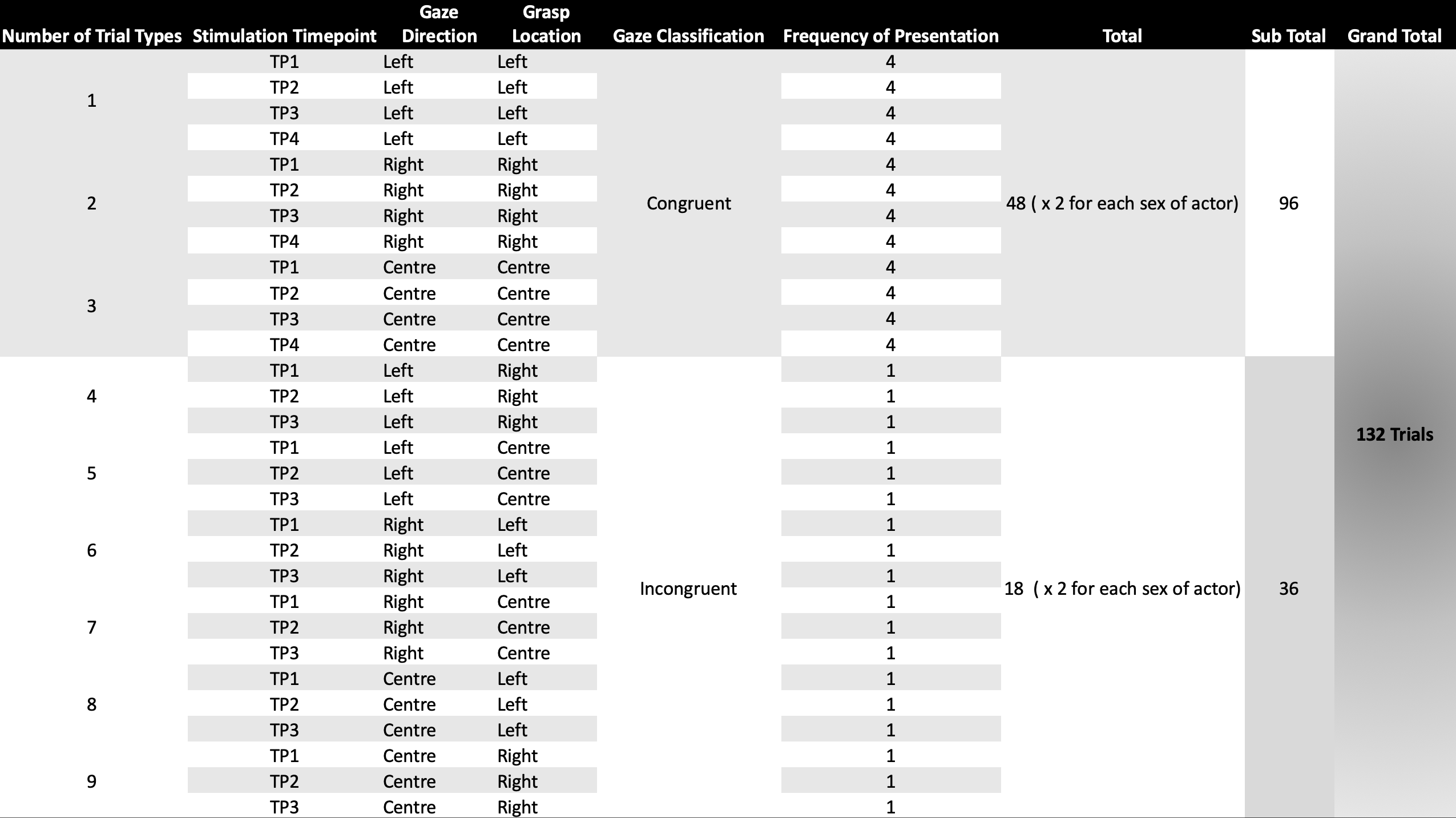
Figure II.* Trial-type information. Gaze direction indicates the placement of the observed cups (left vs centre vs right). See figure 2 below.

**
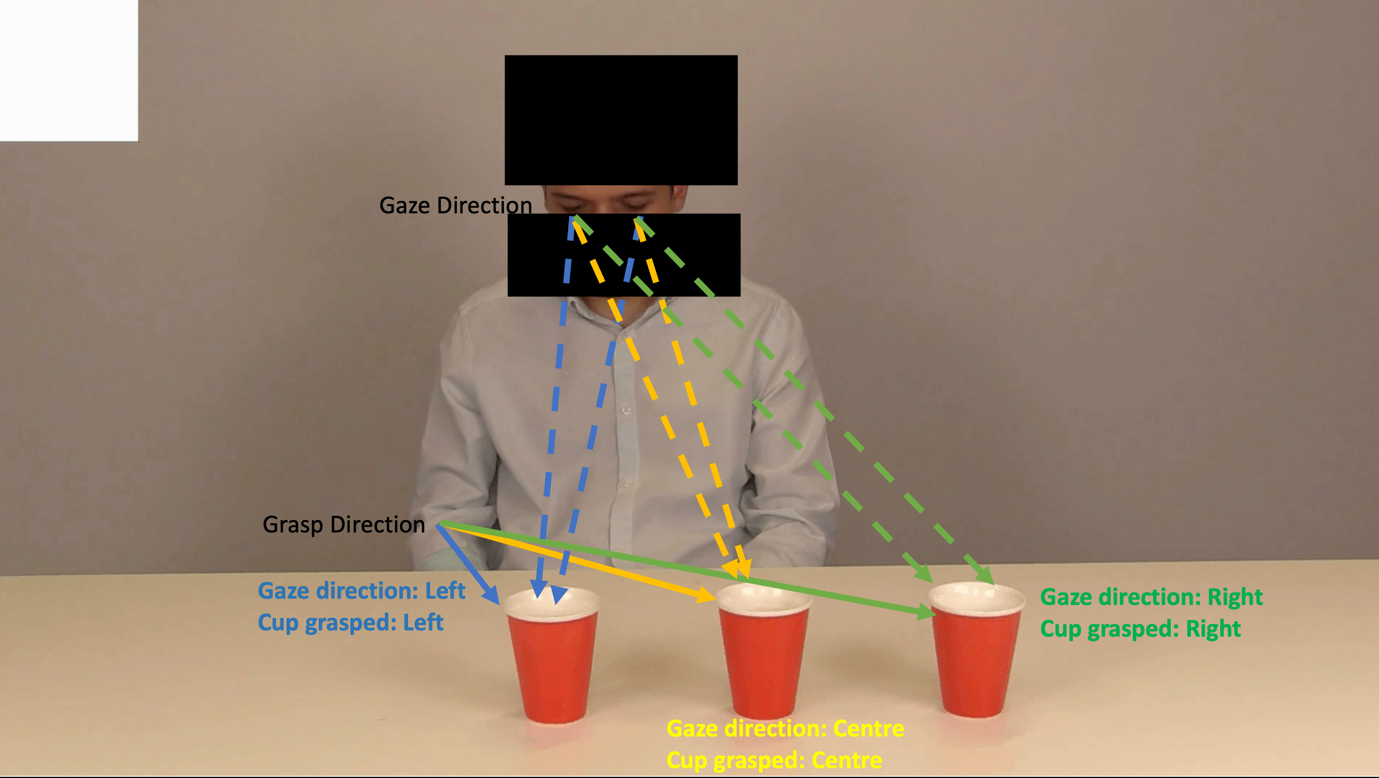
**

*Figure III*. Visual depiction of condition types: congruent. The blocks in images displayed here have been added to de-identify actors, and were not present during the task.

*
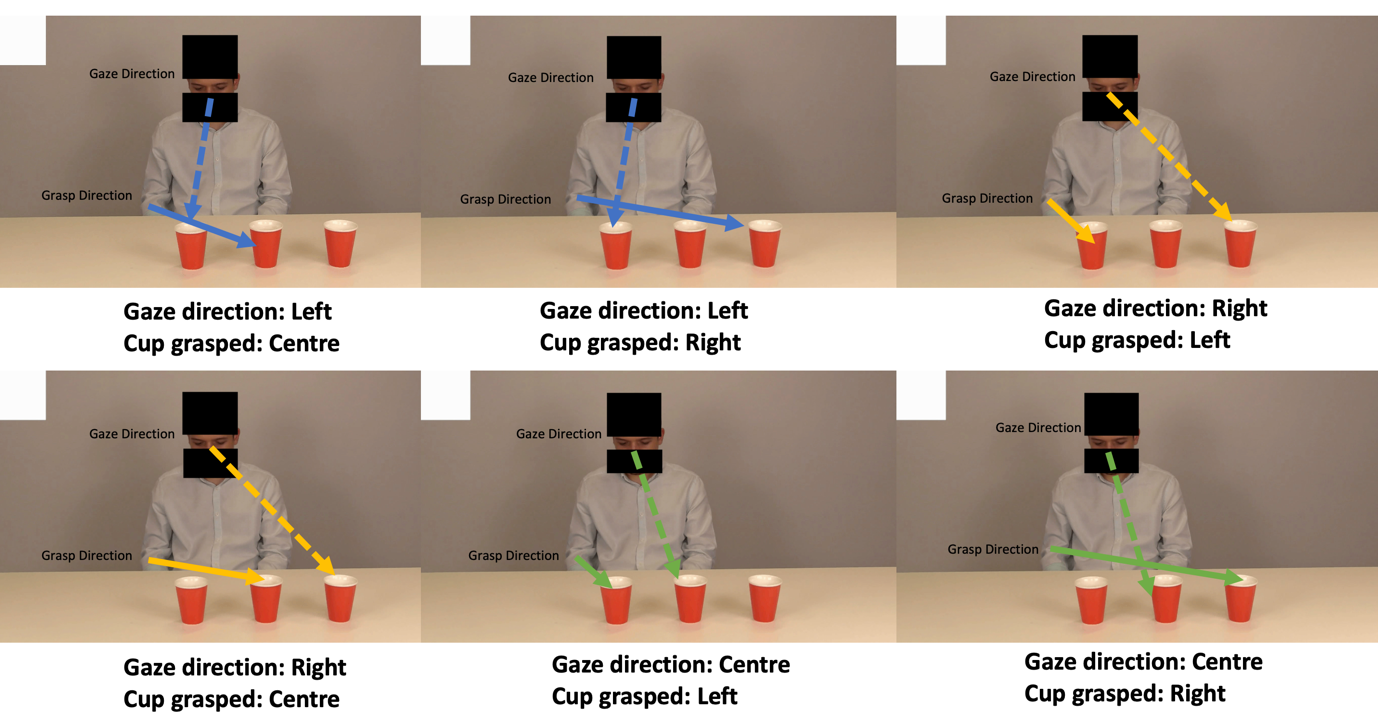
*

*Figure IV*. Visual depiction of condition types: incongruent. The blocks in images displayed here have been added to de-identify actors, and were not present during the task.

**Table 1.** Comparison of IMR between non-normal data and log transformed data.

|  | ***Original (Non-normal) Data*** | | ***Log Transformed Data*** | |
| --- | --- | --- | --- | --- |
| **Main effects** | ***Effect*** | ***p*** | ***Effect*** | ***p*** |
| Change from baseline | 60.35% | <.001 | z = 92.58 | <.001 |
| Block Effect | χ^2^ = 19.85 | <.05 | χ^2^ = 13.79 | <.001 |
| Stim Time effect | χ^2^ = 36.84 | <.001 | χ^2^ = 54.96 | <.001 |
| Interaction with Block | χ^2^ = 4.76 | .571 | χ^2^ = 2.95 | .815 |
| General Fixation Effect | χ^2^ = 1.98 | .739 | χ^2^ = 5.41 | .248 |
| Interaction with Block | χ^2^ = 11.90 | .453 | χ^2^ =17.66 | .126 |
| Biological Effect | χ^2^ = 1.44 | .229 | χ^2^ =1.49 | .222 |
| Interaction with Block | χ^2^ = 0.93 | .818 | χ^2^ = 2.48 | .478 |
| Movement Effect | χ^2^ = 0.01 | .918 | χ^2^= 0.33 | .568 |
| Interaction with Block | χ^2^ = 2.80 | .423 | χ^2^ = 8.17 | .042 |
| Attention Effect | χ^2^ = 0.41 | .523 | χ^2^ = .02 | .880 |
| Interaction with Block | χ^2^ = 0.47 | .925 | χ^2^ = .51 | .916 |
| **3-way Interactions** | *No Interactions* | | *No Interactions* | |
| **Block one only** |  |  |  |  |
| Stim Time effect | χ^2^ = 16.99 | <.001 | χ^2^ = 18.04 | <.001 |
| General Fixation Effect | χ^2^ = 8.38 | .078 | χ^2^ = 6.67 | .154 |
| Biological Effect | χ^2^ = 6.33 | < .05 | χ^2^ = 3.83 | .050 |
| Movement Effect | χ^2^ = 3.54 | .060 | χ^2^ = 3.02 | .082 |
| Attention Effect | χ^2^ = 1.43 | .231 | χ^2^ = 0.31 | .578 |
| **Interactions** | *No Interactions* | | *No Interactions* | |
